# Patellar Tendon Revision Reconstruction Using Transosseous Patella All‐Suture Anchors, Semitendinosus Autograft, and Achilles Allograft

**DOI:** 10.1002/atn2.70151

**Published:** 2026-06-25

**Authors:** Brett P. Salazar, Daniel Yang, Ravi Krishnan, Brent R. Sanderson

**Affiliations:** ^1^ Department of Orthopaedics and Physical Performance University of Rochester Medical Center Rochester New York U.S.A.

## Abstract

Patellar tendon rupture is an uncommon but highly treatable injury, with excellent repair outcomes and low reoperation rates. Chronic patellar tendon ruptures or repair failures pose a clinical challenge, but the success rates of reconstruction with extensor mechanism allograft or autograft are reported to be high in the literature. Still, the reconstruction of chronic patellar tendon ruptures can be technically complex and yield inferior outcomes compared with primary repair. A wide array of operative strategies exists to combat these poor results, ranging from Achilles allograft augmentation to transosseous suture constructs. Our combined revision reconstruction approach employs knotless suture anchors, semitendinosus autograft, Achilles tendon allograft, and circumferential suture cerclage. This surgical technique confers the postoperative benefits of autograft and allograft augmentation while preserving the native patellar tendon tissue.

## Graphical abstract

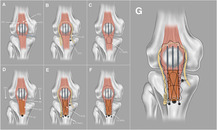

**VIDEO 1:** Technique for patellar tendon revision reconstruction using transosseous patella all‐suture anchors, semitendinosus autograft, and Achilles allograft. A longitudinal incision is made through the previous knee incision scar extending from proximal patella to tibial tubercle. A total of three 2.4 mm transosseous bone tunnels are made within the patella. Three knotless suture anchors are then anchored onto the superior cortical bone of the patella. The semitendinosus autograft is then harvested using an open‐tendon stripper. The semitendinosus tendon is secured just medial to the tibial tubercle at the level of the patellar tendon insertion. It is then shuttled proximally using a Pulvertaft weave technique through the remnant medial retinaculum, passing under the vastus medialis oblique, quadriceps tendon, and vastus lateralis before being shuttled down the lateral aspect of the remnant lateral patellar retinaculum. One limb of a SutureTape is then secured in a locking Krackow fashion along the medial side of the torn patellar tendon and passed through both the knotless loops of the central‐based anchor. Four to 5 additional Krackow whipstitches are placed using the other limb of the SutureTape, and the 2 are tied distally. The free sutures of the double knotless suture anchor are then tensioned to reduce the patellar tendon to its proper patella alignment and height for the extensor mechanism reconstruction. The distal end of the Achilles allograft is fashioned into a bone block on the back table. A guidewire is then placed just distal to the tibial tubercle and used to drill a socket. The bone block is press‐fit into this tunnel and secured using a BioComposite FastThread interference screw. The Achilles tendon is then brought down to overlie the previously tensioned repair and secured to the inferior pole of the patella by interconnecting the medial and lateral anchor knotless mechanisms around the Achilles tendon, creating a suture staple. The knotless suture staple is then tensioned to secure the allograft. Two SwiveLock anchors are then placed on the anteromedial and anterolateral tibia just distal to the tibial tubercle. One limb from each of the medial and lateral anchors is then placed into the SwiveLocks. The semitendinosus autograft is also secured to the lateral SwiveLock anchor. The Achilles tendon and soft tissue repair is completed by flipping the remaining proximal Achilles tendon distally, where it is imbricated with the native patellar tendon using Vicryl sutures. A circumferential relaxing SutureTape is woven and tied around the periphery of our construct, and the remaining Achilles allograft is sutured on top of the final repair to protect from abrasion and serve as additional collagen scaffold for healing. Video content can be viewed at https://doi.org/10.1002/atn2.70151.

Patellar tendon rupture is a rare injury, affecting 0.68 out of 100,000 people annually.^1^ In the setting of complete rupture, operative management in the form of transosseous or suture anchor‐based repair is standard of care.^2^, ^3^, ^4^, ^5^ Acute patellar tendon repair outcomes are often excellent, as indicated by patient reported outcome measures.^4^, ^6^, ^7^ Current literature shows only 2% to 3% of acute repairs require reoperation.^8^, ^9^

In the setting of chronic ruptures or repair failures, extensor mechanism allograft or autograft reconstruction is typically employed.^10^ The success rate of reconstruction in chronic patellar tendon tears is reportedly high.^11^, ^12^ There are a variety of reconstruction techniques for chronic patellar tendon ruptures or primary repair failure, including hamstring tendon autograft and allograft, contralateral bone‐tendon‐bone autografts, collagen augmentation, and Achilles allografts.^9^, ^11^, ^13^, ^14^, ^15^, ^16^, ^17^, ^18^, ^19^, ^20^, ^21^, ^22^

We describe a combined autograft and allograft reconstruction technique in the setting of a previous failed patellar tendon repair. This technique allows for the preservation of existing native tissue while also augmenting the repair with both semitendinosus autograft and Achilles allograft.

## SURGICAL TECHNIQUE

A longitudinal incision is made through the previous knee incision scar from the proximal patella to the tibial tubercle. The patellar tendon tear is identified as well as tears of the medial and lateral retinaculum. The remnant stump of native patellar tendon is identified on the tibial tubercle. The inferior patella is stripped of soft tissue and the bony surface decorticated down to healthy, bleeding bone. A Cobb elevator is used to remove soft tissue adhesions proximally to allow sufficient excursion of the extensor mechanism for reconstruction.

**FIGURE 1:**
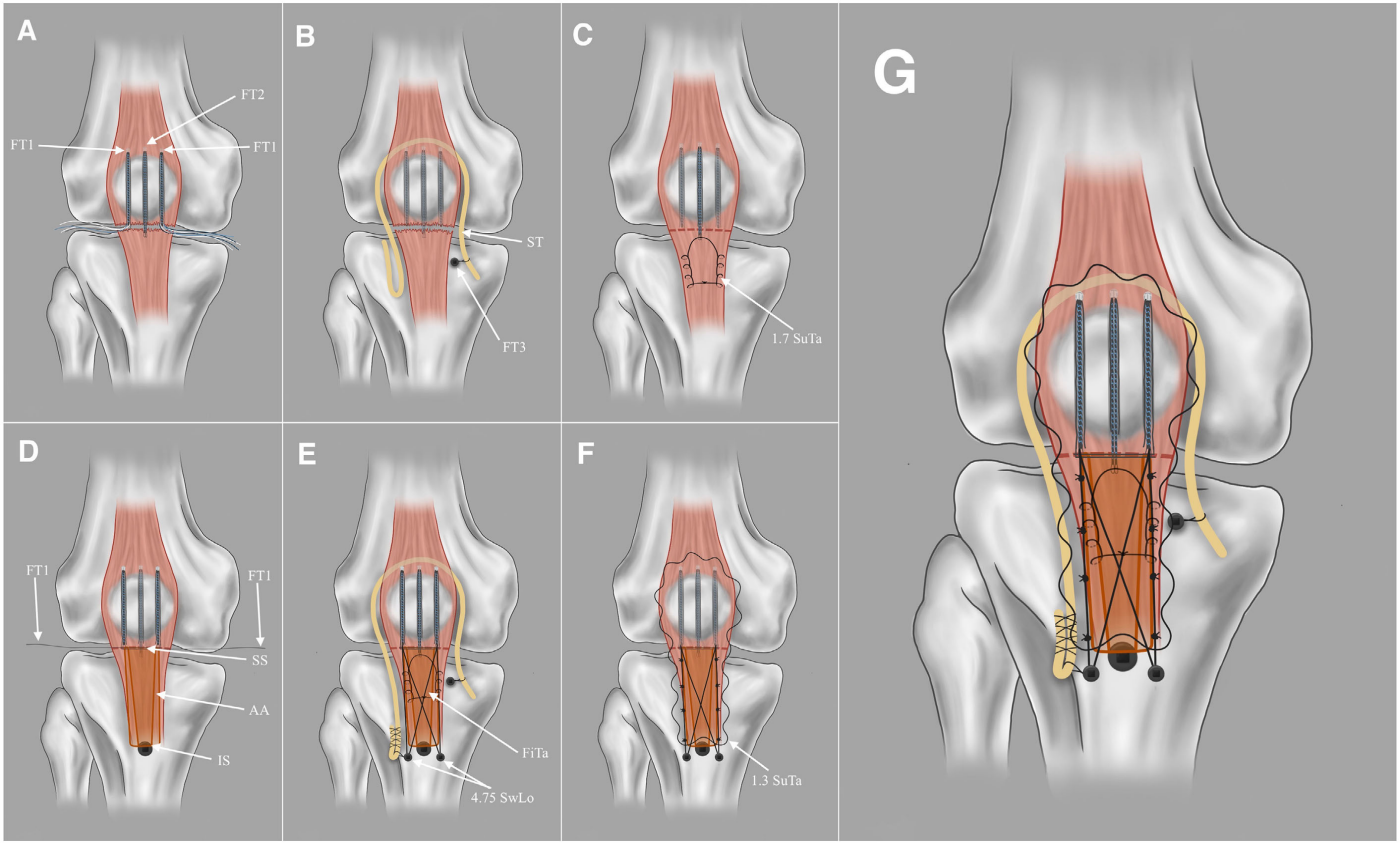
(A‐G) Layers 1 to 6 showing the steps of our patellar tendon repair and reconstruction on a right knee in the supine position. (A) Layer 1: Two 2.6 mm knotless FiberTak RC suture anchors (FT1) are placed through medial and lateral transosseous bone tunnels. A 2.6 mm Knee FiberTak Double Knotless suture anchor (FT2) is placed in the middle transosseous bone tunnel. (B) Layer 2: Semitendinosus autograft (ST) is harvested and woven around the superior patella and secured medially using a 2.6 mm FiberTak hybrid knotless anchor (FT3) suture anchor. (C) Layer 3: The patellar tendon is repaired using a Krackow stitch with 1.7 mm SutureTape (1.7 SuTa) and tensioned using a 2.6 mm Knee FiberTak Double Knotless suture anchor (FT2) through the central bone tunnel. (D) Layer 4: An Achilles allograft (AA) is placed using an interference screw (IS). The medial and lateral suture anchors (FT1) create a suture staple (SS) that the Achilles allograft is folded over. (E) Layer 5: FiberTape sutures (FiTa) from the medial and lateral suture anchors are crossed over the Achilles graft and secured distally using 4.75 mm PEEK SwiveLock anchors (4.75 SwLo). The semitendinosus autograft is tensioned and secured distally. (F) Layer 6: A cerclage 1.3 mm SutureTape (1.3 SuTa) was placed around the periphery of the repair. (G) Complete patellar tendon reconstruction with all layers.

Three 2.4 mm longitudinal transosseous bone tunnels (medial, central, and lateral) are made with a cannulated drill within the patella from distal to proximal, spaced about 1.5 cm apart. A longitudinal split is made through the quadriceps tendon down to the superior pole of the patella in line with each drill hole. A nitinol wire is passed through the cannulated drill and used to shuttle a FiberLink (Arthrex, Naples, FL) through each of the 3 tunnels with the loop exiting proximally. Three suture anchors are first removed from their inserters. Using a FiberLink, the suture limbs of each anchor are shuttled from proximal to distal through one of the bone tunnels. A 2.6 mm knotless FiberTak RC suture anchor (Arthrex, Naples, FL) is placed in the medial tunnel and lateral tunnel. A 2.6 mm Knee FiberTak Double Knotless suture anchor (Arthrex, Naples, FL) is placed in the central tunnel. We lengthen the knotless loops from the knee FiberTak anchor to allow for passage through the length of the patella. The knotless soft tissue anchors are anchored and deployed on the superior cortical bone of the patella. The longitudinal splits in the quadriceps tendon are closed at the end of the procedure, burying the soft tissue anchor to avoid soft tissue irritation. The sutures are each snapped and placed aside for later use (Figure 1A).

**FIGURE 2:**
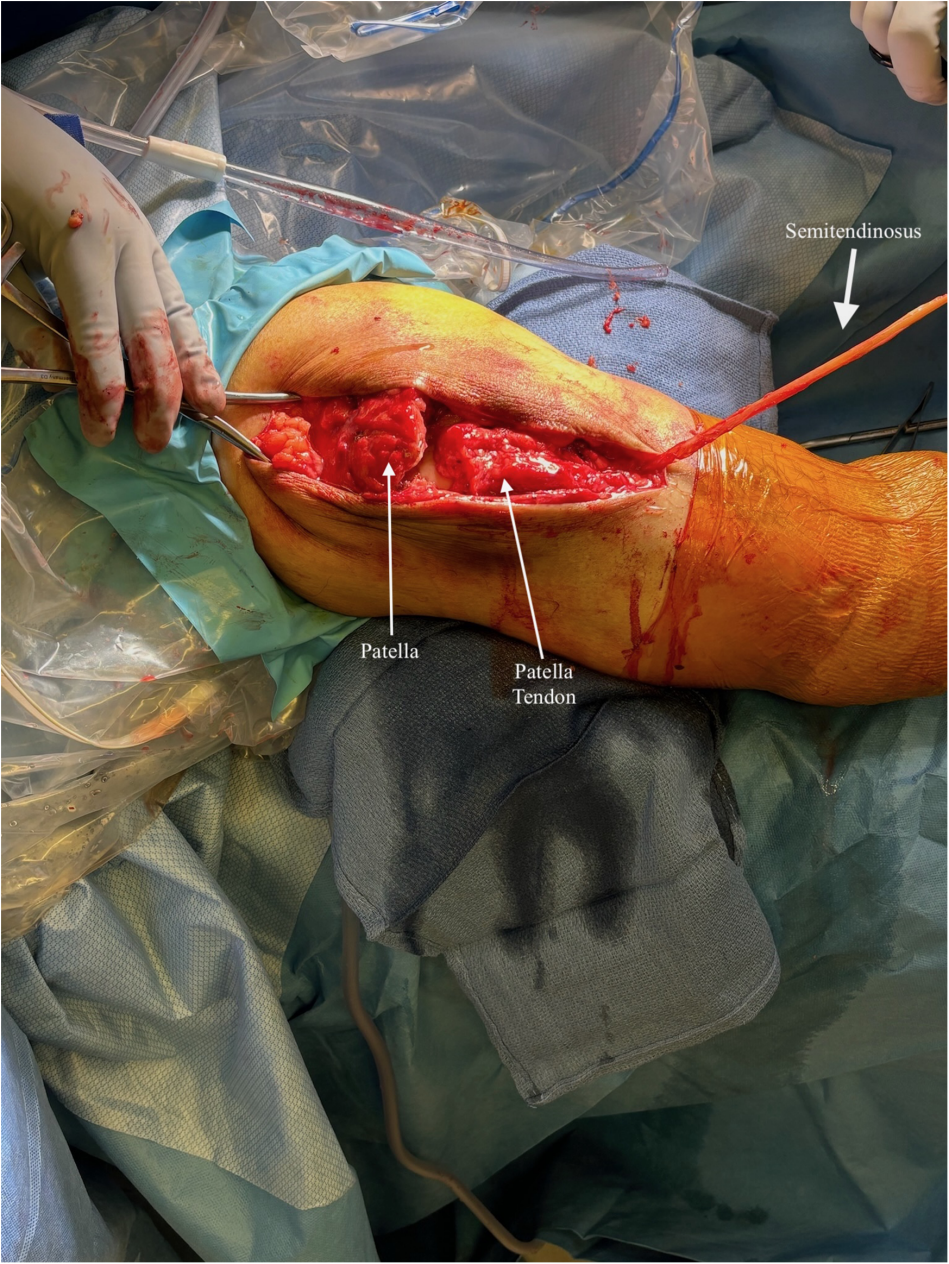
Intraoperative photograph of a right knee harvesting the semitendinosus tendon autograft, leaving it attached distally at the pes anserinus.

The semitendinosus autograft is harvested using an open‐tendon stripper, leaving it attached distally at its insertion at the pes anserinus (Figure 2). Residual muscle is trimmed from the tendon. The tendon is first fed under the suture staple from a 2.6 mm FiberTak hybrid knotless anchor (Arthrex, Naples, FL) placed just medial to the tibial tubercle at the level of the patellar tendon insertion, aligning the graft with the patellar tendon. The tendon is shuttled proximally using a Pulvertaft weave technique through the remnant medial retinaculum, passing under the vastus medialis oblique, quadriceps tendon, and vastus lateralis, and shuttled down the lateral aspect of remnant lateral patellar retinaculum (Figure 1B). The tendon is set aside for later tensioning and fixation on the anterolateral tibia later in the case.

**FIGURE 3:**
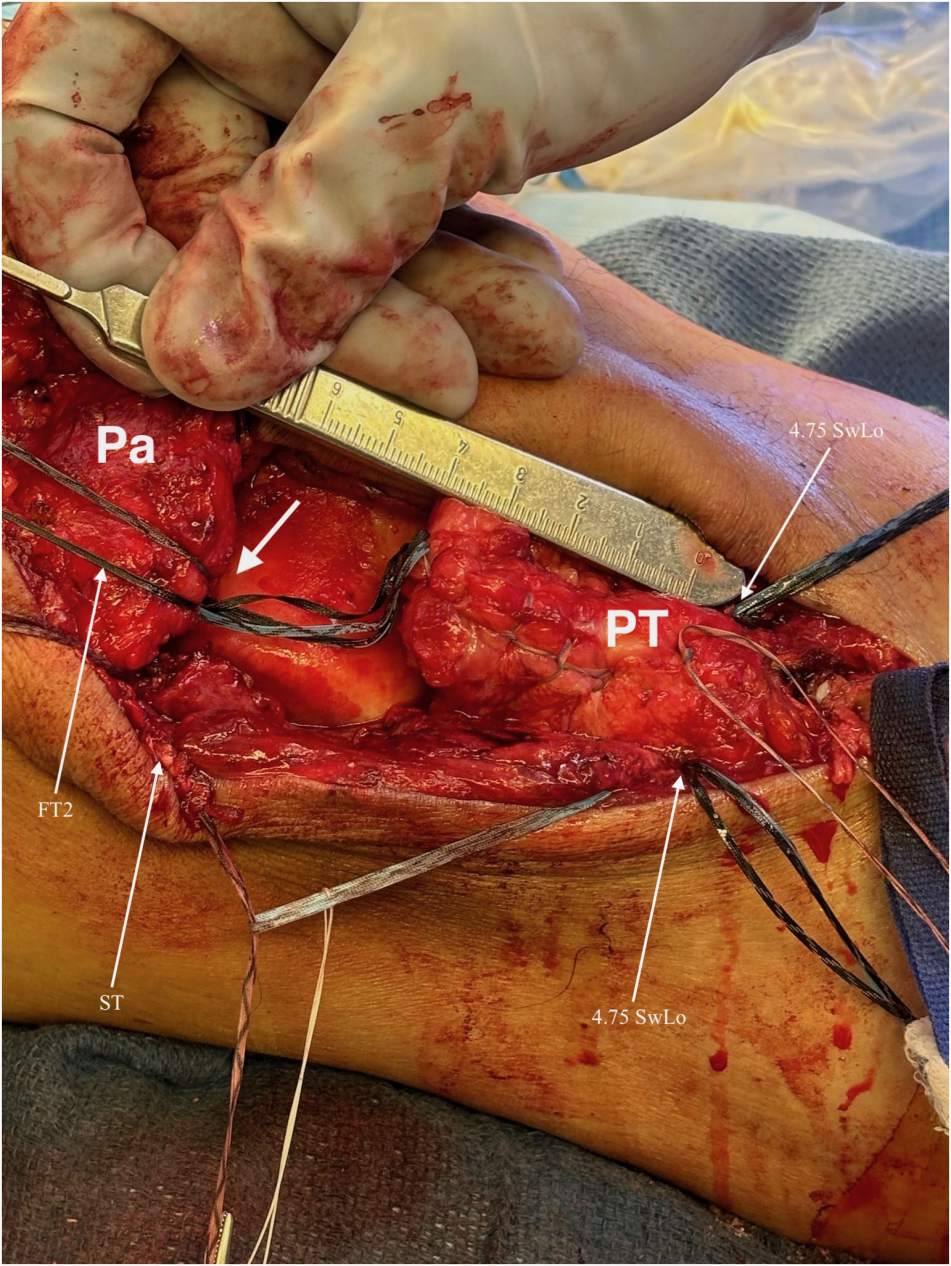
Intraoperative photograph of a right knee showing the 1.7 mm SutureTape passed through the knotless loops of the central‐based 2.6‐mm Knee FiberTak Double Knotless suture anchor (FT2) (arrow) in the patella (Pa), with Krackow whipstitches in the native patellar tendon (PT). Additionally, the semitendinosus autograft (ST) and 4.75 mm PEEK SwiveLock anchors (4.75 SwLo) are visualized, to be secured in later steps.

A free 1.7 mm SutureTape (Arthrex, Naples, FL) is used in locking Krackow fashion along the medial side of the torn patellar tendon remnant, beginning distally and working proximally for a total of 4 to 5 whipstitches. With the needle still attached, this limb of the SutureTape is passed through both the knotless loops of the central‐based anchor. The same limb is used to suture the patellar tendon from proximal to distal, placing 4 to 5 additional whipstitches (Figure 1C). The two 1.7 mm SutureTape limb ends are tied distally, and the knot is buried (Figure 3). Tension is applied to the double knotless suture anchor tensioning limbs to reduce the patellar tendon to its preoperatively templated patella alignment.

The distal end of the Achilles allograft is fashioned into a 9 mm × 22 mm bone block on the back table. A unicortical guide wire is placed just distal to the tibial tubercle from anterior to posterior. A 10 mm‐cannulated reamer is placed over the guidewire and is used to drill a 10 mm by 25 mm socket. The Achilles bone block is press‐fit into this tunnel and secured with an 8 mm × 20 mm BioComposite FastThread interference screw (Arthrex, Naples, FL). The screw is placed on the superior side of the bone plug to secure the bone plug into the tibial tubercle tunnel to allow the tendinous portion to drape anterior over the screw and bone block.

The Achilles tendon is brought proximally to overlie the previous tensioned repair and secured to the inferior pole of the patella by interconnecting the medial and lateral anchor knotless mechanisms (Figure 1D). This creates a suture staple by shuttling the repair limb of the previously placed medial suture anchor under the Achilles allograft using the shuttle suture from the lateral suture anchor (Video 1). This is repeated with the repair limb of the lateral suture anchor being shuttled through the medial anchor. This knotless suture staple is tensioned to secure the allograft at an appropriate length over the patellar tendon. The 2 repair suture limbs are tied to provide additional fixation.

The FiberTape suture ends of the medial and lateral anchors are crossed over the Achilles allograft for suture augmentation. Two 4.75 mm PEEK SwiveLock anchors (Arthrex, Naples, FL) are placed on the anteromedial and anterolateral tibia, just distal to the tibial tubercle. One FiberTape limb from each of the medial and lateral patella anchors are placed in the medial and lateral SwiveLocks. The whipstitch sutures of the semitendinosus autograft are also placed in the lateral SwiveLock anchor. The knee is placed at approximately 20° of knee flexion during anchor placement (Figure 1E).

**FIGURE 4:**
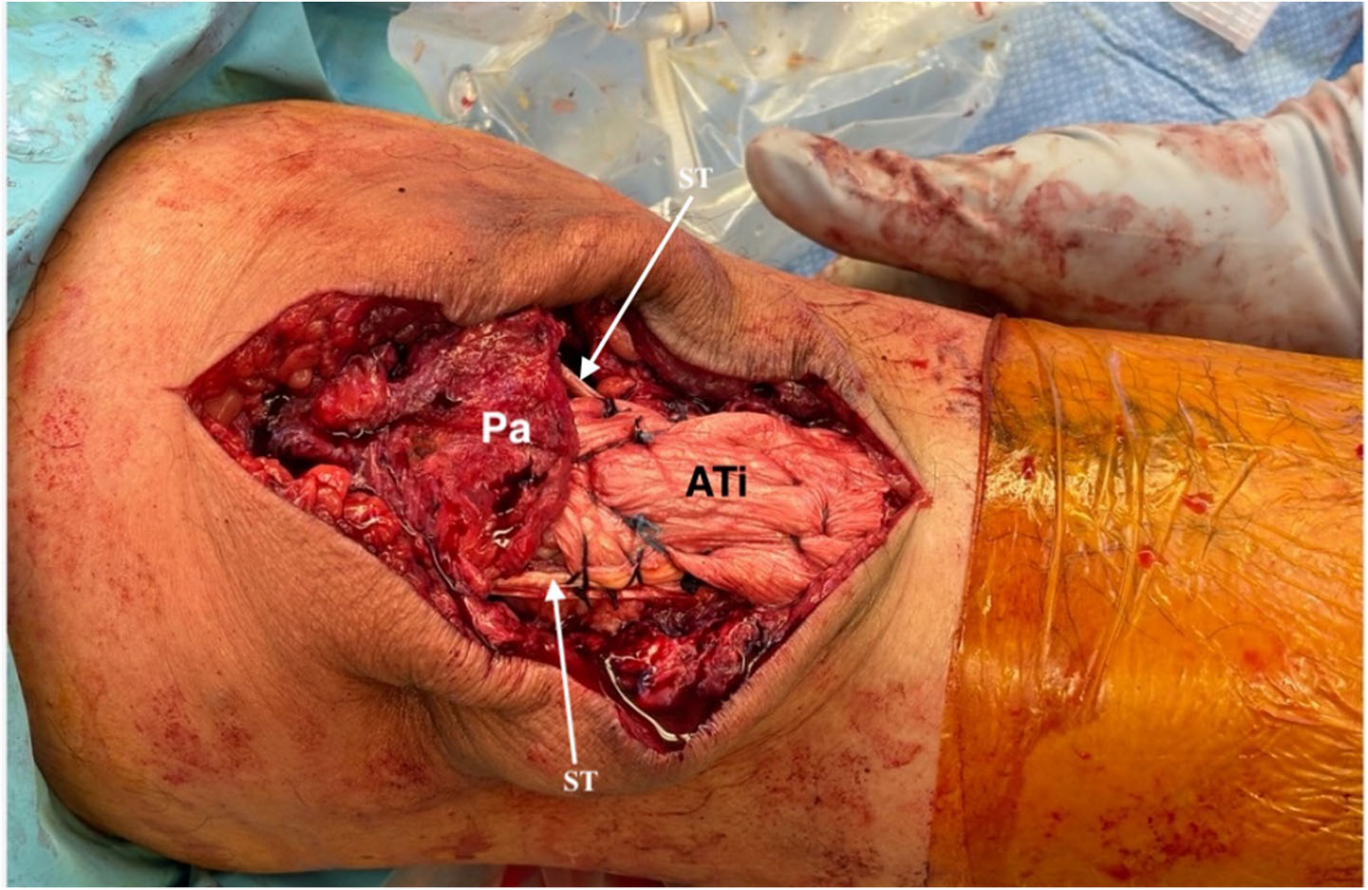
Intraoperative photograph of the right knee patellar tendon reconstruction immediately after Achilles tendon imbrication (ATi) with the native patellar tendon using #1 Vicryl sutures. Similarly, the semitendinosus autograft (ST) is imbricated to the Achilles allograft repair. (Pa, patella.)

The remaining proximal Achilles tendon is flipped distally, where it is imbricated with the native patellar tendon using #1 Vicryl sutures (Figure 4). Any remaining Achilles tendon is resected and soaked in a bowl containing saline and vancomycin.

A 1.3 mm SutureTape “relaxing suture” is passed circumferentially around the patella.^23^ The relaxing suture is passed through the patellar tendon and allograft tissue distally, woven through the medial retinaculum, across through the quadriceps tendon and lateral retinaculum so that it encompassed the repair (Figure 1F). This is tied with the knee in 30° of flexion.

The previously resected Achilles tendon allograft is trimmed to size and sutured over top of the entire construct to serve as a collagen scaffold and avoid suture knot abrasion with the subcutaneous tissue. The retinaculum is repaired with additional 1.3 mm SutureTape, burying the knots. The knee is ranged from 0° to 45° to ensure no gapping at the repair sites. The subcutaneous layers and skin are closed and dressed in standard fashion. A standardized postoperative protocol is followed (Table 1).

**TABLE 1:** Standardized Postoperative Protocol

**Postoperative Period**	**Immobilization/Brace**	**Weight Bearing Status**	**Medications**	**ROM/Physical Therapy**
Week 0‐3	Long leg cast; skin check at Week 1	Touchdown WBAT	Apixaban 2.5 mg BID weeks 0‐2; Aspirin 81 mg BID weeks 2‐3	No ROM (cast)
Week 3‐4	Hinged knee brace locked in extension	WBAT in full knee extension	Aspirin 81 mg BID weeks 3‐4	PT unlocks brace to 0‐15°
Week 4‐6	Hinged knee brace	WBAT in full knee extension	None	ROM progresses to ~30°
Week 6‐12	Hinged knee brace	WBAT in full knee extension	None	ROM increases 15° every 2 weeks
Up to 3 months	Hinged knee brace (progressively unlocked)	WBAT in full knee extension	None	Continued ROM progression with PT

BID, twice daily; PT, physical therapy; ROM, range of motion; WBAT, weight bearing as tolerated.

## DISCUSSION

The reconstruction of chronic patellar tendon ruptures can be technically challenging and results in worse outcomes compared with primary repair.^23^ To combat these poor results, strategies such as Achilles tendon allograft or semitendinosus autograft augmentation, end‐to‐end suture repair with cerclage supplementation, transosseous suture constructs, and suture anchors are all commonly employed in the revision setting.^24^, ^25^, ^26^, ^27^, ^28^, ^29^ Numerous studies have reported the results of these techniques in isolation.^11^, ^13^, ^16^, ^30^ This technique can be used in patients for whom the operating surgeon may worry about repair construct strength and integrity (Table 2).

**TABLE 2:** Advantages and Disadvantages

**Advantages**	**Disadvantages**
Retensionable repair construct: Suture anchors proven to be biomechanically superior with less tendon gapping	Uncertain long‐term efficacy: Case series of patient outcomes needed to evaluate efficacy of these repair
Interference screw Achilles allograft fixation: Shown to result in less tibial trauma and easier patient recovery	Increased operative time: Synthesis of multiple augmentation strategies may prolong operative time, carrying higher risk of complications
Achilles allograft augmentation: Shows increase in postoperative ROM and quadriceps strength while minimizing extensor lag and rerupture rate	Higher resource expenditure: A greater number of implants used may translate to increased cost of materials

Our described technique is a mix of several previously reported techniques. Based on the technique of Stokes et al., we used all‐suture anchors through transosseous tunnels, gaining the advantage of a retensionable repair construct with less gapping while minimizing the traditional complication risks.^31^ The semitendinosus autograft component of our technique draws from techniques described by van der Zwaal et al. and Chen et al.^32^, ^33^ Unlike the 2 Achilles tendon allograft techniques secured with cortical screws previously described by Ginesin et al. and Halvorson et al., we used an Achilles allograft secured with an interference fit screw similar to a technique published by George and Jorgensen, which results in less trauma to the tibia and may allow an easier patient recovery.^34^, ^35^, ^36^

This technique has limitations, namely the lack of reported long‐term patient outcomes (Table 3). Despite this, we believe that our comprehensive repair and augmentation technique is valuable to the growing body of literature surrounding chronic patellar tendon ruptures and should be considered in surgical planning of complex patients with chronic injuries.

**TABLE 3:** Pearls and Pitfalls

**Pearls**	**Pitfalls**
Establish patellar height before allograft placement with central anchor tensioning system: Proper patellar height determines correct length‐tension relationship for subsequent Achilles and semitendinosus augmentation	Technically demanding operation: Surgeons may have to overcome a learning curve in combining multiple repair techniques
Bury the suture anchors deep to quadriceps tendon: Ensures anchors are sitting directly on bone and sealed beneath tendon, minimizing postoperative irritation	Inadequate excursion of proximal extensor mechanism with Cobb elevator: Leads to excess tension on the repair and grafts, risking repair failure or maltracking
Flip proximal Achilles allograft distally over final construct: Provides extra soft tissue layer for improved durability and healing	Poor tibial fixation of Achilles bone block: Achilles construct can loosen and compromise reconstruction

## DISCLOSURES

The author (B.R.S.) declare the following financial interests/personal relationships which may be considered as potential competing interest: B.R.S. reports a relationship with Arthrex that includes educational grants; reports a relationship with Miach Orthopaedics, Stryker, Arthrex, and Prodigy Surgical Distribution, that includes support for attending meetings and/or travel. The other authors (B.P.S., D.Y., R.K.) declare that they have no known competing financial interests or personal relationships that could have appeared to influence the work reported in this paper.
